# Stress and Burnout in EFL Teachers: The Mediator Role of Self-Efficacy

**DOI:** 10.3389/fpsyg.2022.880281

**Published:** 2022-04-28

**Authors:** Heli Chang

**Affiliations:** Department of Foreign Languages, Dongchang College of Liaocheng University, Liaocheng, China

**Keywords:** teacher self-efficacy, burnout, self-efficacy, mental condition, positive upshots

## Abstract

Within the class environment, particularly in the language learning setting, stress is considered to be the most common mental condition educators experience in their work, and due to the effect of stress on teachers, burnout similarly occurs because English as a foreign language (EFL) teachers periodically experience affective trauma while participating and engaging in their careers. To solve the problem, one must consider teacher self-efficacy, a significant construct that can mitigate the probability of burnout by preventing the occurrence of stress at work that also plays a key role in enhancing positive upshots. To emphasize the impact of teachers’ stress and burnout on the one hand and the mediator role of teachers’ efficacy, on the other hand, this mini-review attempted to study the issue. Finally, some scholastic recommendations are pinpointed.

## Introduction

Possibly everyone can experience the stress phenomenon at work because of a lack of time and resources, or other types of density from co-workers, supervisors, or other structures of the organization. Studies have always shown that stress and burnout are positively associated with higher employee turnover, higher absence, and lower performance (Riolli and Savicki, 2006). Education is regarded as a highly complex and demanding work, needing educators to make immediate decisions within the class (Kyriacou, 2001; Pollard and Collins, 2005). Several researchers in general education and language learning settings defined stress in educators as an annoying emotional experience caused by educational management characterized by conditions like anger, stress, frustration, or depression (Collie et al., 2012; Khan et al., 2012; Liu and Onwuegbuzie, 2012). If these negative experiences persist for a long time, they may cause emotional fatigue and poor performance in work, high fatigue, and a negative viewpoint toward a career (Mariani et al., 2020). Educator stress can severely and negatively affect both the education quality and relationships with learners, deteriorating learners’ academic and interpersonal results (Liu and Onwuegbuzie, 2012). In addition, the essence of language classes, educator—learners interplay, and the difficulties of teaching and learning a language are all cited as the main difficulties EFL educators typically face, which can lead to burnout and quitting jobs (Cavazos, 2009). As stated by Johnson et al. (2005), prolonged stress can cause chronic exhaustion, which is closely related to symptoms of burnout. A significant factor influencing educators’ attrition is burnout, which can make them entirely leave the workplace, as a response to the continued career stressful situation resulting in loss of emotion and motivation and decreased dedication (Maslach and Leiter, 2016). Based on the evidence, burnout is more common among those who offer social and human services like health providing, nursing, and education (Maslach, 2003). However, educators were reported to have the highest level of burnout among the above categories (Skaalvik and Skaalvik, 2014). In a nutshell, the stress of education and burnout are crucial in educator training programs, since it is argued that they are the main causing variables for educator attrition (Kyriacou, 2001; Gallant and Riley, 2014).

Everyone has their unique approach to stress management depending on their unique profile of personality (Bianchi and Brisson, 2019). Each educator has unique personal factors, systems of value, beliefs, and perceptions that largely affect the decisions they make and the activities they perform within the class (Tschannen-Moran and Hoy, 2001). Efficacy beliefs symbolize important perceptions that may keep personnel against the destructive consequences of job stress (Bandura, 2006; Capone and Petrillo, 2012). The basic concept of Bandura’s cognitive social theory is self-efficacy which is a key factor of people’s agility, that is, the power of intentional action to change the living setting and environment (Bandura, 2006). Educator efficacy brings up their judgment regarding their functionality to affect learners’ involvement and learning (Hoy, 2004). Undeniably, the teachers’ self-efficacy construct is considered to be a significant feature in academic education (Shaterian Mohamadi and Asadzadeh, 2012) as in the past 20 years, some research has shown that educator efficacy is drastically inspired through educators’ perceptions in their unique teaching context, assessing the resources and support available to them, and the needs in their educational activity (Tschannen-Moran and Hoy, 2001). In the meantime, it is proven that educator efficacy is carefully associated with educator well-being like work involvement, emotional burnout, and satisfaction with a job (Skaalvik and Skaalvik, 2014).

Consequently, many studies were conducted over the past 20 years to determine the relationship between several educator-related variables, namely educator self-efficacy, dedication, resilience, burnout, and satisfaction with a job (Skaalvik and Skaalvik, 2014; Fathi and Derakhshan, 2019; Han and Wang, 2021). Nevertheless, investigations on the relationship between educator efficacy, stress, and burnout are limited, and such studies in the context of EFL are not sufficient. The important issue here is that, to the best of the researchers’ knowledge, there are not enough studies investigating the simultaneous effect of this issue on EFL Chinese educators. Accordingly, the aim of the current review was set to scrutinize the role of the aforementioned issue.

## A Mini Background

### Burnout

The concept of burnout commonly refers to the syndrome related to work originating from the person’s opinions of a major gap among potentials and prospects of victorious performance and a witnessed and way much less pleasant reality (Schaufeli and Taris, 2005); they mostly take place within those whose work needs in-person interaction, along with the need for support like teaching. Scholars indicated that burnout is related to bodily symptoms and depression. Results related to educators` burnout encompass educator attrition, their health problems, and negative outcomes related to learners (Bianchi and Brisson, 2019). Upshots related to teachers’ burnout embrace teacher attrition, their health problems, and destructive consequences in students (Brunsting et al., 2014; Wang and Guan, 2020). It is contended that burnout is composed of three-dimensional interdependent elements, such as emotional exhaustion, depersonalization, and poor accomplishment (Maslach and Leiter, 2016). Emotional exhaustion begins the vital fundamentals of burnout and an individual’s sense of emotive emptiness due to job stress, arguments, embarrassments, and job burden (Skaalvik and Skaalvik, 2014). Depersonalization was labeled as a route of apathy and disinterest in a person’s work as Depersonalized people are motivated to respect their work and those they are engaged in their career negatively (Maslach and Leiter, 2016).

### Stress

The definition of stress refers to any negative emotion-provoking situation and educators experience stress when they are angry, nervous, anxious, exhausted, or depressed (Kyriacou, 2001). Several factors are among those causes of teacher stress that are classified by the previous studies. For instance, the learners’ misbehavior, improper communication with colleagues or parents, short time, weak working situation, dissatisfaction with salaries, and a large number of learners are the major causes (Clipa and Boghean, 2015). For teachers, the three main stressors are personal, interpersonal, and organizational (Prilleltensky et al., 2016). Personal stressors consist of educator self-efficacy and capability while interpersonal stressors represent educator’s relationship with supervisor, learners, parents, and colleagues, while organizational stressors contain the culture of the student, school management, and regulations made by the government (Prilleltensky et al., 2016). Negative feelings that educators experience need to be controlled properly. Unmanaged stress will lead to burnout and quitting the profession.

### Self-Efficacy

In his study, Bandura (2006) discovered a self-efficacy mechanism affecting behavior. As a quantity of self-related awareness, self-efficacy is a key component of the motivational process that affects the levels of motivation. High-efficacy educators are willing to work hard to find the solution to their own challenges; this is while low-efficacy educators truly avoid academic challenges to handle them. In addition, low-efficacy educators have to dedicate large amounts of energy to alleviate emotional stress and combat withdrawal that increases emotional fatigue and depersonalization (Bandura, 2006; Han and Wang, 2021). Consequently, educators can protect themselves against occupational burnout through self-efficacy as a personal resource factor (Zheng and Shen, 2013). Studies indicate that the efficacy of personal teaching and career burnout are negatively related, which is also the case between the overall instructional efficacy and career burnout (Jiang et al., 2007). Bandura’s study tackles the source of the emergence of self-efficiency where humans as active beings can regulate themselves and modify their behavior, rather than being passive humans controlled by unfamiliar conservational powers or inner movements. They can take an active part in changing themselves and take control of events and phenomena through their actions. According to Bandura, self-efficacy increases individuals’ motivation and cognitive resources, which is also a factor in managing the specific incident. Confidence in one’s own self-efficacy is the foundation of motivation, a better life, and individual achievement in all fields of life (Simarasl et al., 2010).

## Final Remarks

The bond between the educational system and learners are educators, and their stress can result in a loss of enthusiasm for engagement in their classes that subsequently bring about burnout. Conversely, teachers’ efficacy highly determines the success of the educational programs in the academic system. Undoubtedly, one can take a large step toward reaching higher education objectives by examining this factor (Barari and Barari, 2015). Thus, upon knowing the qualifications required for the career, developing the efficacy of the educators is obtained. Self-efficacy generally alludes to believing in one’s capability to handle stresses and challenges as Woolfolk and Davis (2006) also indicate that educators’ self-efficacy influences lots of positive factors during class time, including low-stress levels, student achievement, and long careers. As a result, the development process of teacher self-efficacy can begin with the educator training program designed by an educator trainer. Self-efficacy refers to an individual’s response to a variety of difficult situations focusing on people’s behavioral and emotional responses to a variety of situations. Gramstad et al. (2013) support the role of efficacy on burnout, arguing that people with high self-efficacy are relatively less close to experiencing burnout because of active coping tactics.

In addition, teachers with a low level of self-efficacy experience higher burnout levels and are more likely to quit their careers. In times of stress and burnout, beliefs of self-efficacy are significant personal resources (Emmanuel, 2019). In view of these factors aforementioned, it is of paramount importance to develop EFL/ESL teachers’ resilience against these negative emotions (Xue, 2021; Wang et al., 2022). Indeed, lower self-efficacy beliefs are associated with higher rates of affective burnout and depression (García-Carmona et al., 2019). The review is noteworthy for school managers to know that self-efficacious teachers were commonly more about regulating stress and hesitation and they are more determined in completing their job with no stress. Accordingly, school faculty administrators must arrange for a dynamic and energetic condition for their teachers to preserve their self-efficacy and consequently their well-being that can enhance an encouraging and effective language class.

The result is significant for EFL teachers to be more self-efficacious in dealing with burnout since those who encounter burnout do not make much effort to grow the theoretical achievement of their learners and consider that they cannot confidently impact scholars. Likewise, teachers with more self-efficacy are inclined to be superior at organizing more malleable during obstacles, and more balanced and supportive to their students. In the same vein, high self-efficacious teachers are frequently motivated to cope with stress and hesitations and they try more to justify their tasks and duties without any compression.

Additionally, this mini-review proves that educators’ self-efficacy was closely related to burnout aspects and that the implementation of various tactics helped them cope with stress and achieve objectives (Yost et al., 2019). Therefore, as EFL educator trainers are assured regarding the importance of the role of self-efficacy in alleviating stress and even diminishing it, carrying out various classes for EFL educators are recommended to enhance their self-efficacy, so the review is highly noteworthy for them and they are encouraged to train competencies associated with manipulating ones’ stressors in an attempt to control their level of burnout, too. Likewise, academic managers and administrators play a significant role in ensuring that these educators meet their learning needs while in a class. Moreover, these additional training programs on stress management can be conducted *via* workshops to support educators to become more concerned in the training process and arranging their job policies.

More empirical research can be conducted especially qualitative types through meditative instruments to get more perception about the role of teacher efficacy, stress, and burnout in the language situation. More empirical and academic inquiries are compulsory to scrutinize the mediator contribution of teachers’ efficacy in lessening teachers’ stress and burnout.

## Author Contributions

The author confirms being the sole contributor of this work and has approved it for publication.

## Conflict of Interest

The author declares that the research was conducted in the absence of any commercial or financial relationships that could be construed as a potential conflict of interest.

## Publisher’s Note

All claims expressed in this article are solely those of the authors and do not necessarily represent those of their affiliated organizations, or those of the publisher, the editors and the reviewers. Any product that may be evaluated in this article, or claim that may be made by its manufacturer, is not guaranteed or endorsed by the publisher.
